# *Anopheles rufipes* implicated in malaria transmission both indoors and outdoors alongside *Anopheles funestus* and *Anopheles arabiensis* in rural south-east Zambia

**DOI:** 10.1186/s12936-023-04489-3

**Published:** 2023-03-16

**Authors:** Kochelani Saili, Christiaan de Jager, Onyango P. Sangoro, Theresia E. Nkya, Freddie Masaninga, Mwansa Mwenya, Andy Sinyolo, Busiku Hamainza, Emmanuel Chanda, Ulrike Fillinger, Clifford M. Mutero

**Affiliations:** 1 International Centre of Insect Physiology and Ecology (Icipe), P.O. Box 30772-00100, Nairobi, Kenya; 2 University of Pretoria Institute for Sustainable Malaria Control, School of Health Systems and Public Health, University of Pretoria, Pretoria, South Africa; 3 Mbeya College of Health and Allied Sciences, University of Dar es Salaam, Mbeya, Tanzania; 4 World Health Organization, Lusaka, Zambia; 5 National Malaria Elimination Centre, Lusaka, Zambia; 6 World Health Organization, Regional Office for Africa, Brazzaville, Congo

**Keywords:** *Anopheles rufipes*, *Anopheles funestus*, *Anopheles arabiensis*, Vector-control, Entomological inoculation rate, Zambia

## Abstract

**Background:**

The primary malaria vector-control interventions, indoor residual spraying and long-lasting insecticidal nets, are effective against indoor biting and resting mosquito species. Consequently, outdoor biting and resting malaria vectors might elude the primary interventions and sustain malaria transmission. Varied vector biting and resting behaviour calls for robust entomological surveillance. This study investigated the bionomics of malaria vectors in rural south-east Zambia, focusing on species composition, their resting and host-seeking behaviour and sporozoite infection rates.

**Methods:**

The study was conducted in Nyimba District, Zambia. Randomly selected households served as sentinel houses for monthly collection of mosquitoes indoors using CDC-light traps (CDC-LTs) and pyrethrum spray catches (PSC), and outdoors using only CDC-LTs for 12 months. Mosquitoes were identified using morphological taxonomic keys. Specimens belonging to the *Anopheles gambiae* complex and *Anopheles funestus* group were further identified using molecular techniques. *Plasmodium falciparum* sporozoite infection was determined using sandwich enzyme-linked immunosorbent assays.

**Results:**

From 304 indoor and 257 outdoor light trap-nights and 420 resting collection, 1409 female *Anopheles* species mosquitoes were collected and identified morphologically; *An. funestus* (n = 613; 43.5%), *An. gambiae *sensu lato (*s.l.*)(n = 293; 20.8%), *Anopheles pretoriensis* (n = 282; 20.0%), *Anopheles maculipalpis* (n = 130; 9.2%), *Anopheles rufipes* (n = 55; 3.9%), *Anopheles coustani s.l.* (n = 33; 2.3%), and *Anopheles squamosus* (n = 3, 0.2%). *Anopheles funestus *sensu stricto (*s.s*.) (n = 144; 91.1%) and *Anopheles arabiensis* (n = 77; 77.0%) were the dominant species within the *An. funestus* group and *An. gambiae* complex, respectively. Overall, outdoor CDC-LTs captured more *Anopheles* mosquitoes (mean = 2.25, 95% CI 1.22–3,28) than indoor CDC-LTs (mean = 2.13, 95% CI 1.54–2.73). Fewer resting mosquitoes were collected with PSC (mean = 0.44, 95% CI 0.24–0.63). Sporozoite infectivity rates for *An. funestus, An. arabiensis* and *An. rufipes* were 2.5%, 0.57% and 9.1%, respectively. Indoor entomological inoculation rates (EIRs) for *An. funestus s.s*, *An. arabiensis* and *An. rufipes* were estimated at 4.44, 1.15 and 1.20 infectious bites/person/year respectively. Outdoor EIRs for *An. funestus s.s.* and *An. rufipes* at 7.19 and 4.31 infectious bites/person/year, respectively.

**Conclusion:**

The findings of this study suggest that *An. rufipes* may play an important role in malaria transmission alongside *An. funestus s.s.* and *An. arabiensis* in the study location.

**Graphical Abstract:**

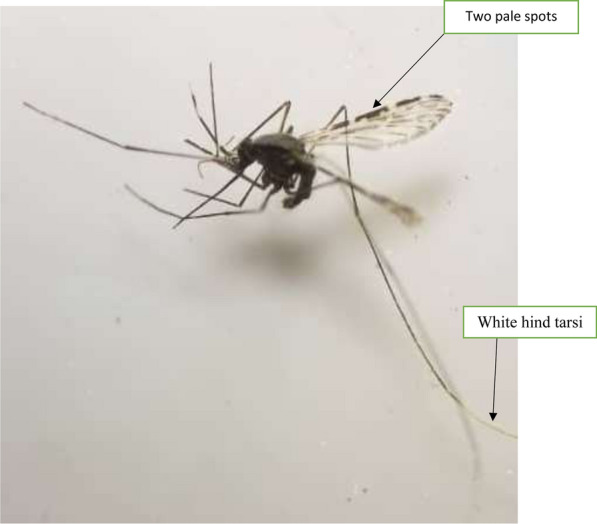

## Background

Malaria is endemic throughout Zambia, where it continues to be a major public health concern. In 2018, Zambia reported a national average malaria parasite prevalence of 9.1% in children under the age of five years [1, 2]. While this signifies progress compared to previous years (2010: 16.0%, 2012:14.9% and 2015:19.4% [1, 3]), this progress is not uniform across the country. In the southern regions, i.e., Lusaka and Southern provinces, malaria incidences have steadily decreased to less than 1% [1]. However, the disease remains intractable in the northern and eastern regions where parasite prevalence can be as high as 30% in children under the age of five years [1]. This is despite high coverages of primary vector-control interventions, namely indoor residual spraying (IRS) and long-lasting insecticidal nets (LLINs) [4–8]. The 2018 nationwide malaria indicator survey indicated that in the southern regions, more than 83% of households had at least one LLIN or had received IRS the previous year. Coverages were higher in the northern and eastern regions; approximately 94% of households had at least one LLINs or had received IRS [1, 2].

The high malaria prevalence has been attributed, in part, to the development of insecticide resistance to commonly used insecticides for malaria vector control [4, 8–10]. Resistance to carbamates, pyrethroids and the organochlorine DDT has been reported in multiple sites in Zambia in the primary malaria vectors *Anopheles funestus* and *Anopheles gambiae *sensu stricto (*s.s*.) [9, 11–14]. Insecticide resistance undermines the continued efficacy offered by both LLINs and IRS by reducing mosquito susceptibility to the insecticides used in the two vector-control methods [15]. Further, behavioural resistance, such as outdoor vector biting and resting behaviour to avoid contact with insecticides, such as the increased exophagy observed in *An. funestus* [16, 17], poses a threat to malaria control and elimination efforts. And whilst increased vector-control interventions have led to a population decline of the primary vectors *An. funestus* and *An. arabiensis* [18, 19], this suppression has sometimes led to a proportionally increased role in malaria transmission by secondary vectors, such as *Anopheles squamosus* and *Anopheles coustani s.l.* [20–23]. In the Southern and Northern provinces of Zambia, *An. coustani s.l.* and *An. squamosus* exhibited anthropophilic tendencies with a high human blood index [23, 24] and were found harbouring malaria parasites [21, 25]. In the Eastern province, Lobo et al. [22], found a larger than expected number of sporozoite infected *An. coustani s.l.* mosquitoes. As many of the secondary vectors are exophilic and exophagic [26], they may have minimal contact with insecticides sprayed on the inside walls of houses or impregnated in LLINs. Subsequently, *An. coustani* s*.l., An. squamosus* or other secondary vectors may evade current vector-control interventions and thus sustain residual malaria transmission after the main endophilic and endophagic vectors have been reduced by IRS and/or LLINs [26, 27].

In recent years, Nyimba district in Eastern province Zambia has benefitted from increased vector-control interventions, primarily IRS and LLINs [13, 28, 29]. The current interventions are primarily intra-domicilliary and target mosquito species that prefer to feed and rest indoors. Thus, malaria vectors which feed, and rest outdoors may elude vector control interventions and be responsible for residual malaria transmission. This phenomenon, therefore, calls for entomological surveillance of all mosquito populations to understand which species might be responsible for transmission and whether, based on their behaviour, they will be sufficiently targeted by current interventions [30]. This study aimed to contribute to the understanding of the species composition of potential malaria vectors and their relative abundance and to determine their sporozoite infectivity and entomological inoculation rates (EIRs) as measures of malaria transmission in rural south-east Zambia and whether they will respond to current interventions.

## Methods

### Study area

**Fig. 1:**
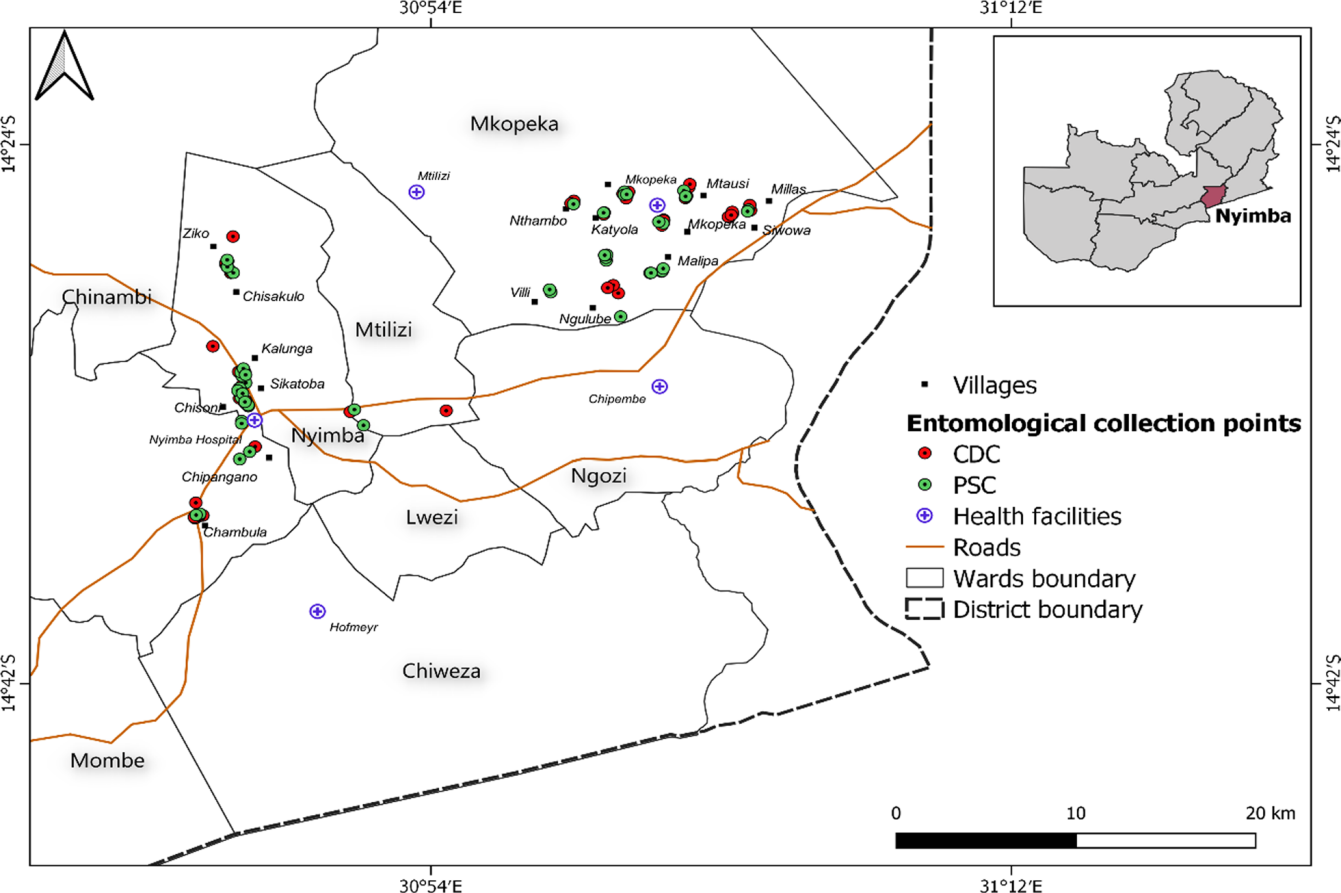
Map of Nyimba district showing the location of households that were used for entomological collection. Insert: Map of Zambia showing the location of Nyimba district

This study was conducted in Nyimba district, located in south-eastern Zambia (Fig. 1) between January-May 2019 and July 2019 to January 2020. Nyimba is predominantly a rural area with an estimated population of 108,637 persons [6]. Geographically, Nyimba district is divided into two parts; the eastern part of the district lies on a plateau whilst the western is in the Luangwa River valley. It shares an international boundary with Mozambique [31]. Nyimba district experiences three distinct seasons. Warm and wet from December to April; cool and dry winter from May to August and, hot and dry from September to November. Malaria transmission is perennial with a reported incidence rate of 467 cases per 1000 persons per year as of 2018 for the entire district [District Health Information System [DHIS]). Malaria transmission peaks after the rainy season between March and May [1].

Two neighbouring health facility catchment areas were selected for this study: Mkopeka and Nyimba Urban (Fig. 1). In 2018 Mkopeka and Nyimba Urban had malaria incidence rates of 414 and 161 cases per 1000 persons/year respectively (Nyimba District Medical Office [DMO]). The houses in the study area were largely of two types: traditional mud or fire brick walls and grass thatched roof and mud or fire brick walls with metallic roofs.

IRS is the frontline vector-control intervention with annual spraying done since 2009 [28]. Starting 2014, IRS had been conducted using blanket application of the organophosphate, pirimiphos-methyl (PM) between the years 2013 and 2018 [13, 28, 32]. In this district LLIN distributions were only done in 2014 and 2018 [33]. However, starting 2019, continuous distribution of LLINs through antenatal care (ANC) clinics and school-based distribution continued as per national guidelines. During the study period, no IRS was conducted in the study area.

### Adult mosquito collection

Longitudinal mosquito surveys were conducted between January-May 2019 and July 2019 to January 2020. No collections were made in June 2019 due to logistical challenges. Households in Mkopeka and Nyimba Urban were enumerated, mapped and each household individually assigned a unique identification number. From the household list generated, 60 houses were randomly selected to serve as sentinel houses for entomological surveillance. Twenty-five served as sentinel houses for Centre for Disease Control and prevention light traps (CDC-LTs; Model 512, John W Hock, Florida, USA); 10 were in Nyimba Urban and 15 in Mkopeka. Another 35 houses were used for pyrethrum spray catches (PSC); 15 in Nyimba Urban and 20 in Mkopeka [13, 34]. The houses were spread across 20 villages. Each village had a minimum of two sentinel houses, 50 m apart, with one house serving for CDC-LT collections and another serving for PSC collections. At least 15 villages had three houses with two for PSC collections.

Mosquito collections were undertaken both indoors and outdoors using CDC-LTs. On each night of collection, two CDC-LTs were deployed per household; one inside and another outside. For indoor collections, the CDC-LT was set up between 18:00 and 06:00 h by hanging the trap, with its entrance 1.5 m above the floor and about 1.5 m away from the feet of a person sleeping under a treated mosquito net [35]. For outdoor collections, the CDC-LT was hung 5–10 m from where the family would usually sit to eat and/or spend evenings before going to bed. This distance allows for the effective range for CDC-LT whilst preventing inhabitants from acting as unprotected bait [36]. The trap was switched on at 18:00 h and switched off at 06:00 h. Both indoor and outdoor CDC-LTs, collections were made in five nights to complete the 25 houses. For each house, collections were made once per month.

Indoor mosquito resting densities were estimated monthly using pyrethrum spray collections (PSC; Mortein Energy ball®, Reckitt Benckiser) [40]. During each collection, the number of people who slept in the house the previous night and bed net use were made were recorded. PSC collections were made monthly in each of the sentinel houses. Five houses per day were sprayed, requiring 7 days to complete.

### Morphological identification of mosquitoes

All collected mosquitoes were morphologically identified [37] and the physiological status of each female was noted as either unfed, fed or gravid. All morphologically identified *Anopheles* mosquitoes were then individually placed in clearly labelled 1.5 ml microcentrifuge tubes containing silica gel desiccant (Fisher Scientific) and cotton wool and stored for molecular analysis. All culicine mosquitoes were counted and discarded.

### DNA extraction and PCR amplification for species identification

DNA was extracted using a modified salt extraction method [38]. Members of the *An. funestus* group (n = 236; 38.5%) and *An. gambiae* complex (n = 110; 37.5%) were further identified to sibling species level by polymerase chain reaction (PCR) [39–41]. Specimens that did not amplify on either the Gambiae-PCR or Funestus-PCR were confirmed using the internal transcribed spacer-2 ribosomal-DNA polymerase chain reaction i.e., ITS2 PCR. The ITS2 PCR technique targets the ITS2 region of nuclear ribosomal deoxyribonucleic acid (rDNA) to produce amplicons of varying band sizes depending on the mosquito species [21, 40, 44, 45]. In each month of collection, a subset of between 25–60% of the total collected female mosquitoes per species separated by collection method was targeted for species identification by PCR. In months where less than 10 mosquitoes were collected, all were subjected to species identification through PCR.

### Blood meal analysis

Blood meal analysis was performed on blood-fed *An. funestus* (n = 81), *An. gambiae* s*.l.* (n = 33) and *An. rufipes* (n = 7). PCR analysis was used to detect and identify host blood from 121 mosquito abdomens from which DNA was extracted using the multiplex PCR assay [38] which targeted the cytochrome b region of the hosts mitochondrial DNA [38].

### Detection of *Plasmodium falciparum* infection in mosquitoes

A random subsample, by sampling method and month of collection of female *An. funestus* (n = 360/613; 58.7%), *An. gambiae s.l.* (n = 174/293; 59.4%), *An. pretoriensis* (n = 72/282; 25.5%), *An. rufipes* (n = 42/55; 76.3%), *An. coustani s.l.* (n = 18/33; 54.5%) and *An. squamosus* (n = 3/3; 100%) mosquitoes were tested for *P. falciparum* circumsporozoite proteins (CSPs) using sandwich enzyme-linked immunosorbent assays (ELISA) [46]. To avoid false CSP positives common in zoophilic species the ELISA lysates were heated [47]. Sporozoite infectivity was determined separately for mosquitoes caught indoors and outdoors.

### Statistical analyses

All data were entered and stored into an Excel spreadsheet (Microsoft Office 2018) and exported to open-source statistical software R version 3.51 [48] for analysis. Descriptive statistics namely mean catches per trap per night and proportions of mosquitoes caught per sampling method per catchment area were used to summarize the data. Species-specific mean catches were calculated by dividing the total number of mosquitoes caught by the number of trap-nights. The human blood index (HBI), sporozoite infectivity rate (SIR) and entomological inoculation rate (EIR) were calculated as a measure of malaria transmission intensity using the following formulae.

#### Human blood index (HBI)

The human blood index (HBI) was calculated as the proportion of mosquitoes fed on human blood meals out of the total mosquitoes that successfully amplified for blood meals [49].

${\mathrm{Human\,Blood\,Index}}=\frac{Number\,of\,mosquitoes\,with\,human\,blood}{Total\,number\,of\,mosquitoes\,amplified\,for\,blood\,meal}$ Mixed (human + domestic animal) blood meals were added to the number of human blood meals when calculating the HBI.

#### Sporozoite infectivity rate (SIR)

Sporozoite infectivity rate (SIR) is defined as the proportion of *Anopheles* mosquitoes with sporozoites in their salivary glands to the total number of mosquitoes examined for sporozoites [50]. Sporozoite infectivity was determined separately for each species. This was determined using the following formula:

$\mathrm{Sporozoite\, infectivity\, rate}=\frac{Number\, of\, mosquitoes\, with\,sporozoites}{Number\, of\, mosquitoes\, examined}$ Sporozoite infectivity rates were determined separately for indoor (PSC and CDC-LTs) and outdoor (CDC-LTs only) collection methods and were species-specific. The Pearson’s Chi-square tests were used to evaluate the difference in proportions and infectivity rates at an α = 0.05 level of significance.

#### Entomological inoculation rate (EIR)

Entomological inoculation rate (EIR) is defined as the number of infectious bites per person per unit time, usually expressed per year or month [51]. Species-specific EIR was calculated based on the mean number of female *Anopheles* mosquitoes caught per trap/night, without adjusting for room occupancy [10, 50]. Annual EIR was calculated separately for indoors and outdoors using the formula:

$$EIR= SIR \times \frac{\#\,of\,mosquitoes\,collected\,by\, CDC-LT}{ \# of\,CDC-LT\,trap\,nights} \times365\,days$$

For PSC collections, EIRs was calculated using the formula described in [52].

***EIR*** = Human Biting Rate (HBR) x SIR × 365 days where SIR as defined above and the human biting rate as shown below.

$$HBR= HBI \times \frac{Number\,of\,blood-fed\,mosquitoes}{ Number\,of\,occupants\,on\,night\,of\,collection}$$

## Results

### Species composition of *Anopheles* mosquitoes

The sampling design of this study resulted in an overall 304 indoor and 257 outdoor CDC light trap-night collections. Less frequent outdoor CDC-LTs collections were due to the rainy season when heavy rains would interfere with trapping. A total of 420 resting collections were done using the pyrethrum spray catch (PSC) method. The average number of human occupants during PSC collections was three.

A total of 1409 female *Anopheles* mosquitoes were collectively sampled in 977 collections. Overall, seven species were identified morphologically. The *An. funestus* group (n = 613; 43.5%) represented the predominant malaria vectors in the study area followed by *An. gambiae s.l.* (n = 293; 20.8%). Other species were *Anopheles pretoriensis* (n = 282; 20.0%), *Anopheles maculipalpis* (n = 130; 9.2%), *An. rufipes* (n = 55; 3.9%), *An. coustani s.l.* (n = 33; 2.3%), and *An. squamosus* (n = 3, 0.2%). Table 1 summarizes the species composition and mean collections per sampling method per night. Only eight male *Anopheles* mosquitoes were collected: *An. gambiae* s.l. (n = 3) and *An. pretoriensis* (n = 5). At the same time 2052 female culicine mosquitoes were collected.

**Table 1:** *Anopheles* species composition and mean collections per sampling method in the study area

Species	Overall	CDC LT-IN		CDC		PSC	
				LT-OUT			
	N	n	Mean (95% CI)	n	Mean (95% CI)	n	Mean (95% CI)
*An. funestus* group	613	331	1.09 (0.92–1.25)	140	0.55 (0.46- 0.65)	142	0.34 (0.19–0.42)
*An. gambiae s.l*	293	167	0.55 (0.38–0.71)	107	0.42 (0.35–0.49)	19	0.04 (0.02–0.06)
*An. pretoriensis*	282	82	0.27 (0.15–0.39)	183	0.71 (0.46–0.97)	17	0.04 (0.01–0.07)
*An. maculipalpis*	130	53	0.17 (0.06–0.29)	74	0.29 (0.22–0.36)	3	0.01 (0–0.01)
*An. rufipes*	55	6	0.03 (0.02–0.04)	47	0.18 (0.14–0.22)	2	0.004
A*n. coustani*	33	8	0.03 (0–0.05)	25	0.10 (0.08–0.11)	0	0
*An. squamosus*	3	1	0	2	0.01 (0–0.02)	0	0

*CDC* Centers for Disease Control and Prevention, *LT* Light Trap, *PSC* Pythrerum Spray Catches, *IN* Indoor *OUT* Outdoor

**Fig. 2:**
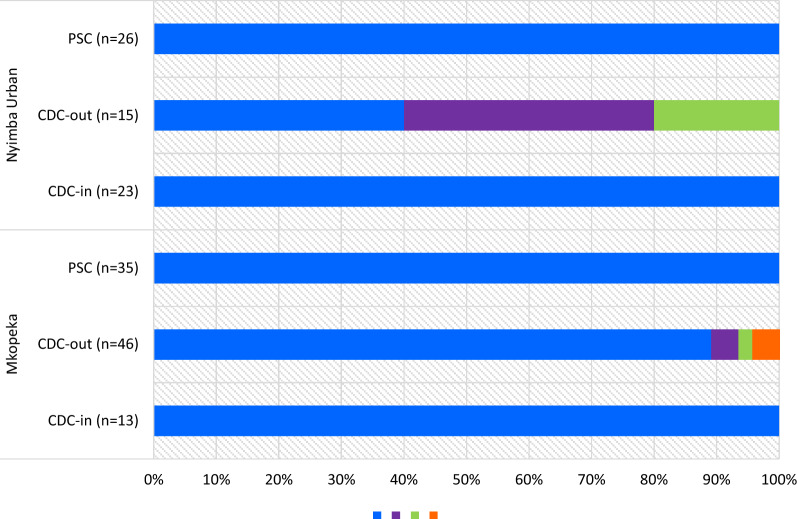
Proportions of species within the *Anopheles funestus* group in the two study areas. The numbers in parentheses indicate the total number of specimens that successfully amplified per collection method per study site

Polymerase chain reaction was performed on a random subsample of 236 (38.5%) of all collected female *An. funestus* mosquitoes. Of these, 158 specimens successfully amplified. A total of 74 specimens did not amplify and four gave non-specific amplification on the ITS2-PCR (n = 2, 700 base pairs and n = 2, 900 bp). Overall, collections from both sites revealed the predominant species found was *An. funestus* sensu stricto (*s.s.*) (n = 144/158; 91.1%); PSC (n = 61/61), indoor CDC-LT (n = 36/36) and outdoor CDC-LT (n = 47/61). There was a significantly higher occurrence of *An. funestus s.s.* in indoor versus outdoor traps (χ^2^ = 7.73, *df* = 1, *P* = 0.03). Other species identified within the *An. funestus* group were *Anopheles leesoni* (n = 8; 5.1%), *Anopheles parensis* (n = 4; 2.5%) and *Anopheles vaneedeni* (n = 2; 1.2%)*. Anopheles leesoni, An. parensis* and *An. vaneedeni* amplified from specimens caught only outdoors. Figure 2 shows the different proportions of species within the *An. funestus* group per sampling method per site.

Polymerase chain reaction (PCR) was performed on a random subsample of 110 (37.5%) female *An. gambiae s.l.* mosquitoes. Of these 100 successfully amplified. Eight did not amplify and two gave non-specific amplifications on the ITS2-PCR (n = 2, 280 bp) upon further analyses.

**Fig. 3:**
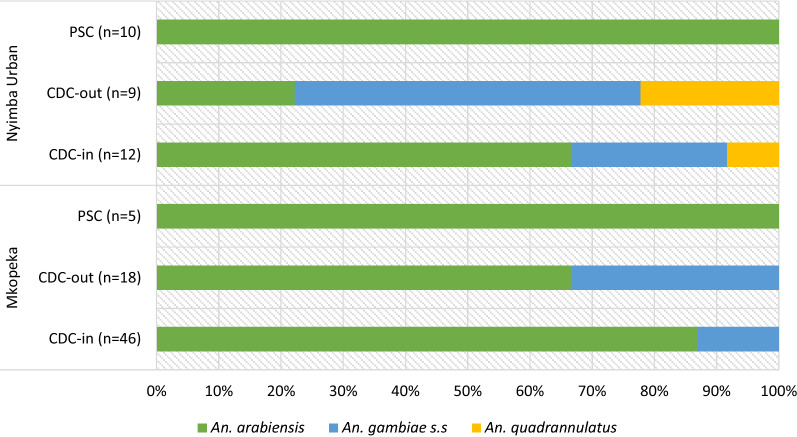
Proportions of species within the *Anopheles gambiae* complex in the two study areas. The numbers in parentheses indicate the total number of successfully amplified specimens per collection method per study site

Within the *An. gambiae* complex, the predominant species was *An. arabiensis* (n = 77; 77.0%); PSC (n = 15/15), indoor CDC-LT (n = 48/58) and outdoor CDC-LT (n = 14/27). *Anopheles gambiae s.s.* (n = 20; 20.0%) and *Anopheles quadriannulatus* (n = 3; 3.0%) were the two other species within this complex in the study area. No *An. gambiae s.s.* were found in PSC with few occurring in indoor (n = 9/61) and outdoor (n = 11/27) CDC-LT collections. Likewise, no *An. quadriannulatus* were collected using PSC with few collected in indoor (n = 1/61) and outdoor (n = 2/27) CDC-LT collections. Figure 3 shows species composition and proportions within *An. gambiae s.l.* per collection method and separated by study site.

### Indoor and outdoor host-seeking and resting collections

Similar numbers of host-seeking *Anopheles* mosquitoes were trapped with light traps outdoors (mean = 2.25, 95% CI 1.22–3.28) and indoors (mean = 2.13, 95% 1.54–2.73) per trap. Fewer mosquitoes were collected per PSC trap night (mean = 0.44, 95% CI 0.24–0.63).

At the species level, more host-seeking mosquitoes of the *An. funestus* group were trapped using indoor CDC-LTs (95% CI 0.92–1.25) per night per house than outdoors (mean 0.55; 95% CI 0.46–0.65) (Table1). Indoor resting densities of *An. funestus* group were slightly lower with a mean of 0.31 (95% CI 0.19–0.42) per house. Only 23.2% of all collected female *An. funestus* mosquitoes (n = 142/613) were caught resting indoors with most of these blood-fed (n = 123/142, 87.6%).

The mean number of *An. gambiae s.l.* mosquitoes trapped with indoor CDC-LTs (mean = 0.55, 95% CI 0.38–0.71) per night per house was slightly higher than collected outdoors (mean = 0.42, 95% CI 0.35–0.49) (Table 1). Only 6.5% of all collected female *An. gambiae s.l*. mosquitoes (n = 19/293) were caught resting indoors with most of these being blood-fed (n = 16/19, 84.2%).

The 503 other anopheline specimens, included the species *An. pretoriensis, An. maculipalpis, An. rufipes, An. coustani s.l.* and *An. squamosus*. Most of these were caught outdoors (n = 318/503, 63.2%) rather than indoors (n = 150/503, 29.8%). Taken together, a larger proportion of these specimens were outdoor host-seeking (χ^2^ = 21.1, df = 4, *P* < 0.01). Few of the other anopheline specimens were caught resting indoors (n = 22/503, 4.4%) with zero blood-fed.

### Blood meal sources

Of the 121 blood-fed mosquitoes analysed, only 18 (14.9%) amplified successfully. Of these, 13 blood meals were from humans and three had mixed human-goat blood meal host (Table 2). The overall human blood index from resting collections and CDC-LT collections both indoors and outdoors was found to be 0.89. Due to the small sample size of mosquitoes that amplified on the blood meal analysis, these results are interpreted with caution.

**Table 2:** Blood meal sources of *Anopheles* mosquitoes per sampling method

Method	*Anopheles* species	# analysed	Human	Mixed: human/goat	Dog	Unamplified	Human blood index
PSC	*An. funestus*	40	3	1	0	36	1.00
	*An. gambiae*	14	2	0	0	12	1.00
CDC LT indoors	*An. funestus*	20	3	1	0	16	1.00
	*An. gambiae*	10	2	0	0	8	1.00
CDC LT outdoors	*An. funestus*	21	3	1	0	17	1.00
	*An. gambiae*	9	0	0	0	9	0.00
	*An. rufipes*	7	0	0	2	5	0.00
Total		121	13	3	2	103	0.89

### Sporozoite infectivity and entomological inoculation rates

A total of 360 (58.7%) female specimens of the *An. funestus* group were tested for the presence of *P. falciparum* circumsporozoite protein (*Pf* CSP). Of these, nine mosquitoes tested positive for sporozoites giving an overall sporozoite infectivity rate of 2.5%. The nine sporozoite infected mosquitoes came from samples collected in February 2019 (n = 3), March 2019 (n = 2), July 2019 (n = 1) and January 2020 (n = 3). All sporozoite infected mosquitoes were *An. funestus s.s.* Other species within the *An. funestus* group, namely *An. leesoni, An. parensis* and *An. vaneendeni* tested negative for *P. falciparum* sporozoites.

A total of 174 (59.4%) female *An. gambiae s.l.* mosquitoes were tested for the presence of the *Pf* CSP*.* One tested positive giving an overall sporozoite infectivity rate of 0.57%. The sporozoite infected mosquito was *An. arabiensis* trapped in March 2019. The other members within the *An. gambiae* complex namely, *An. gambiae s.s.* and *An. quadriannulatus* tested negative for *P. falciparum* sporozoites.

Other anopheline mosquitoes, namely *An. pretoriensis* (n = 70/282; 24.8.0%), *An. coustani s.l*. (n = 17/33; 51.5%), *An. rufipes* (n = 33/55; 94%) and *An. squamosus* (n = 3/3; 100%) were analysed for *Pf-*CSP. Three *An. rufipes* specimens tested positive for sporozoites, giving an overall sporozoite infectivity rate of 9.1% for *An. rufipes* (Table 3). The three sporozoite infected *An. rufipes* were trapped indoors using CDC-LTs in February 2019 (n = 1) and outdoors using CDC-LTs in March and February 2019 (n = 2) in the Mkopeka study sites. The morphological identification of the *An. rufipes* mosquitoes was confirmed using the ITS2-PCR, resulting in an amplification of 500 bp. In all the above, heating the ELISA lysate did not change the *Pf*-CSP positive result.

**Table 3:** Annual EIR estimation based on CDC-LT and PSC catches for *An. arabiensis*, *An. funestus s.s* and *An. rufipes* mosquitoes

Method	Species	# assayed	Sporozoite positive	Proportion of mosquitoes infected (SIR)	EIR (ib/p/yr)
CDC-LT Indoors	*An. funestus* group	179	2	0.01	4.44
	*An. gambiae s.l*	91	1	0.01	1.15
	*An. rufipes*	6	1	0.17	1.20
CDC-LT Outdoors	*An. funestus* group	83	3	0.04	7.19
	*An. gambiae s.l*	83	0	0.00	0.0
	*An. rufipes*	27	2	0.07	4.31
PSC	*An. funestus* group	98	4	0.05	1.19

The species-specific estimated indoor and outdoor annual EIR based on CDC-LT catches for *An. arabiensis*, *An. funestus s.s.* and *An. rufipes* mosquitoes is shown in Table 3. Indoor EIRs for *An. funestus* s.s*,* and *An. arabiensis* were estimated at 4.44 and 1.15 infectious bites per person per year (ib/p/y), respectively. Indoor EIR for *An. rufipes* in the study area was estimated at 1.20 ib/p/y. Outdoor EIR for *An. funestus s.s* and *An. rufipes* were estimated at 7.19 and 4.31 ib/p/y, respectively (Table 3). Only *An. funestus* specimens, collected with PSC, tested positive for sporozoites. Indoor EIRs for *An. funestus s.s,* collected with PSC, was estimated at 1.19 ib/p/y. However, these results are interpreted with caution due to the extremely low number of blood meals that were amplified in the blood meal analysis.

## Discussion

*Anopheles funestus* group made up the majority of anopheline mosquitoes collected in this study. Species identification by PCR further revealed that this group was predominantly made up of *An. funestus s.s.* (henceforth simply referred to as *An. funestus*)*.* This confirms previous reports that describe *An. funestus* as the main driver of malaria transmission in the study area [22, 28, 53]. *Anopheles funestus* is historically highly anthropophilic with strong endophagic and endophilic behaviour [54, 55]. Thus, in the absence of insecticide resistance and/or improved formulations of current insecticides, this species may be controlled by LLINs and IRS [55]. This is supported by the fact that the indoor EIR by *An. funestus* reported in this study (4.4 ib/p/y) was 16 times lower than previously reported in the same location. An EIR of 70.1 ib/p/y was observed between the years 2011–2013 [53]. This decreased EIR may highlight suppression of sporozoite infectivity following increased vector-control interventions, namely LLINs and IRS with pirimiphos-methyl (IRS-PM). These observations are consistent with previous studies conducted in other parts of Zambia which demonstrated the impact of increased IRS-PM and population-wide coverage of LLINs in reducing sporozoite infection rates of *An. funestus* [11, 19]. Similar findings have been reported in neighbouring Mozambique [56], north-western Tanzania [57] and western Kenya [58]. However, that malaria transmission persists, albeit at low levels, shows that these core interventions cannot be deployed solely.

The persistence of malaria has been associated with behavioral changes observed in anopheline mosquitoes. Findings of this study indicate that *An. funestus* may also be transmitting malaria outdoors. In this study, *An. funestus* outdoor EIR, estimated at 7.19 ib/p/y was higher than EIR indoor. The higher outdoor EIR in *An. funestus* may highlight suppression of the highly endophagic species, thereby increasing the proportions of outdoor host seeking mosquitoes [16, 17]. This behavioural modification may be as result of the increased use of LLINs or IRS in the study area [16, 59]. The outdoor malaria transmission described in this study has implications for malaria control and eradication in Zambia and in sub-Saharan Africa. A recent study shows that a 10% increase in outdoor biting would result in 58.2% increase in malaria cases per year on the African continent, assuming a “perfect scenario” of 100% LLINs coverage and zero insecticide resistance [60]. Outdoor biting vectors, thus pose a significant threat to elimination efforts by sustaining malaria transmission. Subsequently, indoor-vector control interventions such as LLINs and IRS alone may not be enough to eliminate malaria [61, 62].

Secondary vectors may also play a role in continued malaria transmission. In this study sporozoite infected specimens of *An. rufipes* were found*.* Similar findings of *An. rufipes* harbouring sporozoites have been reported in southern Zambia [25], Kenya [63], Cameroon [64–66], Burkina Faso [67] and Nigeria [68]. This study thus incriminates *An. rufipes* as a potential malaria vector in rural south-east Zambia [69] with estimated EIRs of 1.20 and 4.31 ib/p/y indoors and outdoors, respectively. The estimated EIR for *An. rufipes* was higher than that of *An. arabiensis,* indicating the need for further studies to investigate the role of secondary malaria vectors in maintaining malaria transmission [26, 70]. Sporozoite infected *An rufipes* mosquitoes were collected during the peak malaria season in Zambia, between February and April [1, 70] when vectors were most abundant. That this species is largely zoophilic and exophagic [25] makes it a threat to achieving malaria elimination as it may evade indoor-centric vector-control interventions [26].

*Anopheles gambiae s.l.*, which was primarily *An. arabiensis,* confirming previous results [71], was found with lower sporozoite infectivity when compared to *An. rufipes*. Thus, in Nyimba district, *An. arabiensis* may be considered a vector of secondary importance when compared to *An. funestus* and *An. rufipes*. This study also confirms previous observations that in cases where *An. arabiensis* and *An. funestus* occur in sympatry, the latter appears to be the more competent malaria vector [55, 72, 73]. Nonetheless, that *An. arabiensis* was found in both indoor and outdoor traps suggest that it can forage both indoors and outdoors thereby making it less amenable to the traditional indoor-based vector-control interventions [19, 74].

The mosquito community in this study included diverse species. Within the *An. funestus* group, were found *An. leesoni, An. parensis* and *An. vaneedeni-* largely zoophilic species [27] all of which tested negative for malaria parasites. Similarly, other members of the *An. gambiae* complex, namely, *An. quadriannulatus* and *An. gambiae s.s.* also tested negative for malaria parasites. However, Lobo et al. [22] found sporozoite infected *An. quadriannulatus, An. pretoriensis* and *An. coustani* from the same study locations. Thus, in this region of Zambia, the vector population plasticity, species diversity and co-occurrence of both primary and secondary vectors with different behaviours, may sustain malaria transmission and calls for more integrated vector-control approaches. Future research should determine the bionomics, morphology, and breeding habitats of potential secondary vectors for a comprehensive understanding of their roles in malaria transmission [21–27]. Additionally, the period (less than a year) and geographical scope of sampling was not extensive and may explain some of the low vector densities observed in this study. More sampling sites are required to establish malaria transmission by *An. rufipes* and other potential secondary vectors. A further limitation of this study was the lack of amplification of some specimens for PCR species identification. This may be attributed to specimen degradation or morphological misidentification, attributed to damaged mosquito specimens. This is common with CDC-LT collections [22]. This calls for improvement in and coupling of morphological identifications with molecular methods of identification. Furthermore, molecular identification was not performed beyond the ITS2 PCR. A two-step procedure for species identification was carried out; first morphological identifications based on morphological keys [37] similar to methods used by Tabue et al.[64] and Awono-Ambene et al. [65]. Second, confirmation of the identification using the ITS2 PCR to ensure that the specimens identified as *An. rufipes* were indeed such. Additional molecular identifications- perhaps by ITS2 gene sequencing to adequately incriminate and identify vectors of malaria [22, 27] should be included in future research.

Findings of this study are limited by several factors. An extremely small number of samples amplified for the blood-meal analyses. Several re-runs were made without success. This might be due to storage conditions. Possibly, DNA of the blood meal host may have been degraded since specimens were stored for several months on silica gel before molecular analysis. Further, mosquitoes may have had incomplete blood meals or the blood meal may have been digested resulting in degradation of host DNA [75]. The successful identification of blood meal hosts by PCR depends on the quality and quantity of the host´s DNA contained in the abdomen of mosquitoes [75]. Yet another possiblity is that mosquitoes fed on hosts other than those included in the primer set e.g., avian-specific primers. Further investigations in blood meal studies in Zambia to document the range of blood meal hosts of malaria vectors are strongly recommended.

## Conclusion

This study confirms earlier reports that *An. rufipes* might be involved in malaria transmission in rural south-east Zambia. Whilst for long, the species has been considered of secondary importance in Zambia due to its largely zoophilic, exophilic and exophagic tendencies, recent successes in vector control require a new evaluation of the remaining vectors. Based on these findings, increased routine entomological surveillance and *Plasmodium* sporozoite infectivity screening for all potential malaria vectors is recommended. Additionally, vector-control interventions should be diversified to include outdoor interventions for improved control and efforts towards malaria elimination.

## Data Availability

The datasets used and/or analysed during the current study are available from the corresponding author on reasonable request.

## Abbreviations

LLINs: Long-lasting insecticidal nets
IRS: Indoor residual spraying
IRS-PM: Indoor residual spraying with pirimiphos-methyl
DMO: District medical office
CDC LTs: Centres for disease control and prevention light traps
PSC: Pyrethrum spray catches
PCR: Polymerase chain reaction
CSP: Circumsporozoite protein
ELISA: Enzyme-linked immunosorbent assays
SIR: Sporozoite infectivity rate
EIR: Entomological inoculation rates
